# Mitochondrial malfunction and atrophy of astrocytes in the aged human cerebral cortex

**DOI:** 10.1038/s41467-023-44192-0

**Published:** 2023-12-16

**Authors:** Alexander Popov, Nadezda Brazhe, Kseniia Morozova, Konstantin Yashin, Maxim Bychkov, Olga Nosova, Oksana Sutyagina, Alexey Brazhe, Evgenia Parshina, Li Li, Igor Medyanik, Dmitry E. Korzhevskii, Zakhar Shenkarev, Ekaterina Lyukmanova, Alexei Verkhratsky, Alexey Semyanov

**Affiliations:** 1 College of Medicine, Jiaxing University, 314001 Jiaxing, Zhejiang Pro China; 2 Shemyakin-Ovchinnikov Institute of Bioorganic Chemistry, Russian Academy of Sciences, Miklukho-Maklaya street 16/10, Moscow, 117997 Russia; 3 Faculty of Biology, Moscow State University, Moscow, 119234 Russia; 4 Department of Neurosurgery, Privolzhskiy Research Medical University, Nizhny, Novgorod 603005 Russia; 5 Institute of Experimental Medicine, St. Petersburg, 197376 Russia; 6 Faculty of Biology, Shenzhen MSU-BIT University, 518172 Shenzhen, China; 7 Faculty of Biology, Medicine and Health, The University of Manchester, Manchester, M13 9PT UK; 8 Achucarro Center for Neuroscience, IKERBASQUE, Basque Foundation for Science, 48011 Bilbao, Spain; 9 Department of Neurosciences, University of the Basque Country UPV/EHU and CIBERNED, Leioa, Spain; 10 Sechenov First Moscow State Medical University, Moscow, 119435 Russia

**Keywords:** Cellular neuroscience, Astrocyte, Neural ageing

## Abstract

How aging affects cells of the human brain active milieu remains largely unknown. Here, we analyze astrocytes and neurons in the neocortical tissue of younger (22–50 years) and older (51–72 years) adults. Aging decreases the amount of reduced mitochondrial cytochromes in astrocytes but not neurons. The protein-to-lipid ratio decreases in astrocytes and increases in neurons. Aged astrocytes show morphological atrophy quantified by the decreased length of branches, decreased volume fraction of leaflets, and shrinkage of the anatomical domain. Atrophy correlates with the loss of gap junction coupling between astrocytes and increased input resistance. Aging is accompanied by the upregulation of glial fibrillary acidic protein (GFAP) and downregulation of membrane-cytoskeleton linker ezrin associated with leaflets. No significant changes in neuronal excitability or spontaneous inhibitory postsynaptic signaling is observed. Thus, brain aging is associated with the impaired morphological presence and mitochondrial malfunction of cortical astrocytes, but not neurons.

## Abstract (web summary)

How aging affects the brain active milieu remains unknown. Here, the authors reveal atrophy and mitochondrial malfunction of astrocytes but not neurons in older human neocortex.

## Introduction

Astrocytes are crucial elements of the brain-active milieu^1,2^ being primarily responsible for homeostatic support and defense of the nervous tissue^3^. In particular, astrocytes act as a metabolic hub, storing glucose, providing metabolic substrates, and supplying neurons with glutamine obligatory for excitatory glutamatergic and inhibitory GABAergic neurotransmission^4^. Physiological brain aging is associated with a decrease in cerebral blood flow^5^ and a decline in glucose metabolism^6^. At the same time, physiological aging with preserved cognitive capacity (in contrast to neurodegeneration) is not associated with profound changes in neuron numbers and morphology^7,8^. Age-dependent changes in astrocytes in the human brain are much less characterized. The numbers of astrocytes in various brain regions are not affected by old age, whereas morphometric studies are somewhat contradictory, with both increases and decreases in the size and complexity of old astrocytes being reported^9^. Most morphological studies on astrocytes employed immunostaining with antibodies against GFAP. The GFAP immunoreactivity, however, reports neither total numbers of astrocytes nor faithfully reveals their morphology^3,10^. Studies of aged astroglia were performed almost exclusively on rodents; human astrocytes are larger and substantially more complex^11^.

Here, we compare age-dependent changes in astrocytes and neurons in human tissue. We show that brain aging is associated with mitochondrial malfunction and atrophy of cortical astrocytes, but not neurons.

## Results

### Mitochondrial function is affected in aged astrocytes but not neurons

**Fig. 1:**
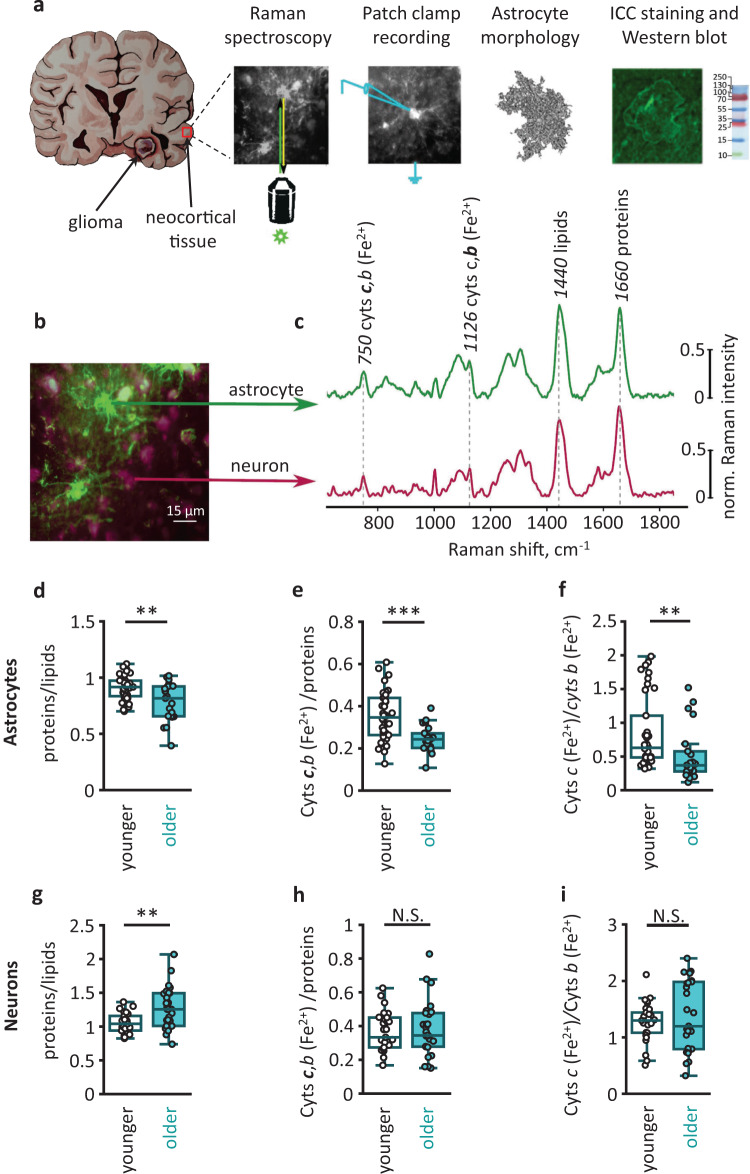
Age-dependent metabolic changes in human astrocytes and neurons. **a** Neocortical tissue was obtained during glioma resection. Neocortical slices were used for Raman spectroscopy, patch-clamp recordings, morphological analysis, immunocytochemical (ICC) staining, and western blot. **b** Double ICC-stained slice, and **c** Raman spectra recorded from GFAP-stained astrocytes (green) and NeuN-stained neurons (burgundy). Spectral peaks marked with Raman shift value and corresponding molecule name. **d** The ratio of proteins to lipids (*P* = 0.009), **e** relative amount of reduced cytochromes (Cyts) of *c*, *b*-types (*P* < 0.001), and **f** the ratio of reduced *c-*type to *b*-type cytochromes (*P* = 0.001) in astrocytes of two age groups (younger adults: *N* = 4 people, *n* = 38 cells; older adults: *N* = 3 people, *n* = 21 cells). **g** The ratio of proteins to lipids (*P* = 0.002), **h** relative amount of reduced cytochromes of *c*,*b*-types (*P* = 0.8) and **i** the ratio of reduced *c-*type to *b*-type cytochromes (*P* = 0.6) in neurons of two age groups (younger adults: *N* = 4 people, *n* = 31 cells; older adults: *N* = 3 people, *n* = 27 cells). Data are shown as box-and-whisker plots where the box is Q1 and Q3 with median, whiskers are the ranges within 1.5IQR. Empty boxes/circles—younger adults, filled boxes/circles—older adults. Two-tailed Mann–Whitney test: ^N.S.^*P* > 0.05, **P < 0.01, ***P < 0.001. Source data are provided as a Source Data file.

We analyzed protoplasmic astrocytes and neurons in the cortical slices prepared from the neocortical tissue (relatively healthy brain tissue removed to gain instrument access to the region of resection) of younger adult (22–50 years old) and older adult (51–72 years old) patients of both sexes subjected to surgical resection of subcortical gliomas or other cancer metastases to the brain (Fig. 1a). First, we investigated the effect of aging on the total protein content and redox state of mitochondria electron transport chain (ETC) in immunocytochemically stained astrocytes and neurons with Raman microspectroscopy (Fig. 1b). This label-free imaging technique allows monitoring vibrational modes of molecules inside cells and tissues providing information about the relative amounts, conformation, and the redox state of detectable molecules^12,13^. In Raman spectra recorded with 532-nm laser excitation, we measured peaks corresponding to lipids, proteins, and reduced cytochromes of *c* and *b*-types in the mitochondrial electron transport chain (ETC) of astrocytes and neurons (Fig. 1b, c). Aging decreased the relative amount of proteins in astrocytes, suggesting a decline in protein synthesis or an increase in the protein degradation rate (Fig. 1d and Supplementary Fig. 1a). Another explanation for this result is an age-dependent accumulation of lipids. The amount of reduced *c*, and *b*-type cytochromes in astrocytes was also lower in older adults reflecting fewer electrons present in mitochondrial ETC at an older age (Fig. 1e and Supplementary Fig. 1b). The more pronounced decline in the reduced *c*-type cytochromes pointed to a higher rate of electron transfer from *c*-type cytochrome to complex IV in astrocyte mitochondria in older adults (Fig. 1f). This suggestion was also supported by a decrease in the amount of reduced *a*-type cytochromes in the ETC complex IV in astrocytes in older adults (Supplementary Fig. 2).

In contrast to astrocytes, the protein-to-lipids ratio increased in neurons of older adults (Fig. 1f and Supplementary Fig. 3a). In addition, we did not observe a significant difference in reduced *c* and *b*-type cytochromes in neurons between age groups (Fig. 1g, h and Supplementary Fig. 3b).

### Aging is associated with astrocytic atrophy

**Fig. 2:**
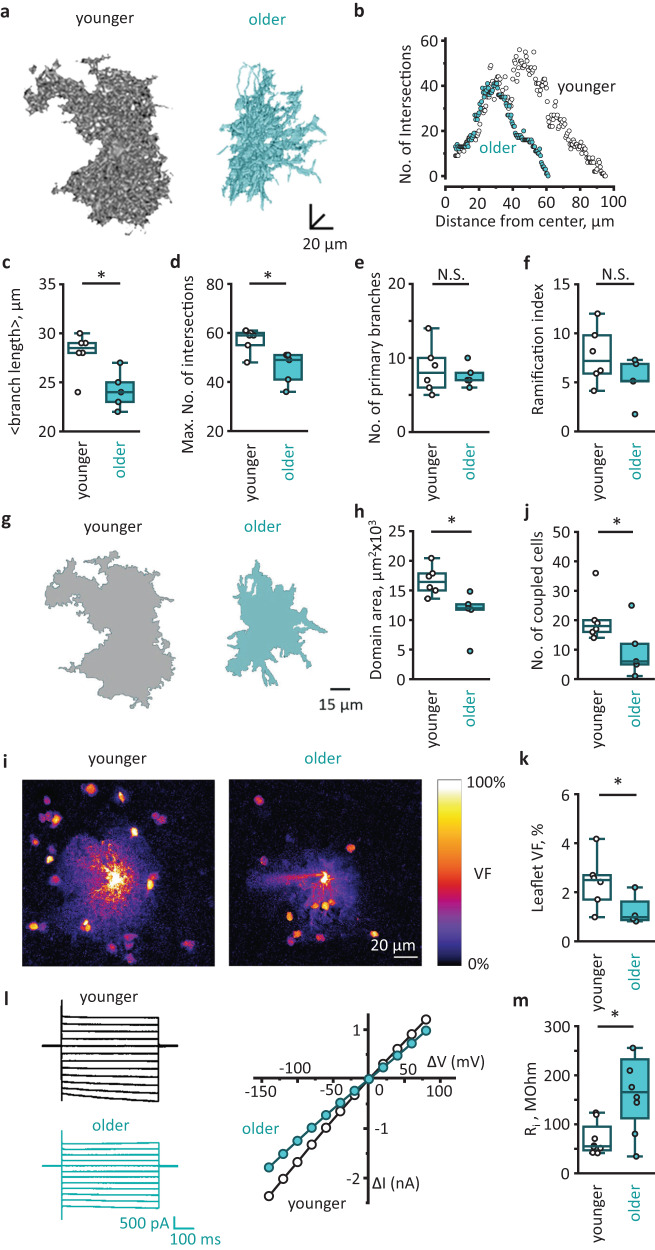
Age-dependent atrophy of astrocytes. **a** Representative three-dimensional (3D) reconstructions of cortical astrocytes loaded with a fluorescent dye (Alexa Fluor 594) through patch pipette in younger (left) and older (right) adults. **b** 3D Sholl analysis of astrocytes shown in (**a**). **c** Mean branch lengths (*P* = 0.03), **d** maximum numbers of intersections (*P* = 0.03), **e** numbers of primary branches (*P* = 0.9) and **f** ramification indexes (*P* = 0.2) in astrocytes of two age groups (younger adults: *N* = 6 people; older adults: *N* = 5 people; cell number *n* = *N*). **g** Astrocytic domain areas obtained as *z*-projections of 3D reconstructions presented at (**a**) in younger (gray) and older (blue) adults. **h** Astrocytic domain areas in two age groups (*P* = 0.01; *N/*n—numbers as above). **i** Astrocytes loaded with Alexa Fluor 594 through patch pipette in younger and older adults. Note fewer neighboring astrocytes loaded through gap junctions in aged astrocytes. The fluorescence intensity was normalized to the fluorescence of soma (100%) to illustrate astrocytic spatial volume fraction (VF) distribution. The scale bar shows color coding for astrocytic VFs from 0 to 100%. **j** Numbers of coupled astrocytes in two age groups (*P* = 0.04; younger adults: *N* = 6; older adults: *N* = 5 people; cell number *n* = *N*). **k** VFs of astrocytic leaflets in two age groups (*P* = 0.03; younger adults: *N* = 6; older adults: *N* = 4 people; cell number *n* = *N*). **l** Representative currents recorded in response to voltage steps (left; black traces—younger adults, blue traces—older adults) and corresponding current–voltage relationships (right) in astrocytes of two age groups. **m** Input resistances (R_i_) of astrocytes in two age groups (*P* = 0.04; younger adults: *N* = 6 people, *n* = 8 cells; older adults: *N* = 7 people, *n* = 7 cells). Data are shown as box-and-whisker plots where the box is Q1 and Q3 with median, whiskers are the ranges within 1.5IQR. Empty boxes/circles—younger adults, filled boxes/circles—older adults. Two-tailed (except for (**j**), where one-tailed) Mann–Whitney test: ^N.S.^*P* > 0.05, *P < 0.05. Source data are provided as a Source Data file.

Astrocytic atrophy was reported in aged mice^14^. However, the results obtained in animals may not always apply to the human brain, especially considering significant differences in astrocytic morphology between humans and rodents^11^. Moreover, the research was done only on male mice, further limiting the study. Here, we analyzed the morphology of cortical protoplasmic astrocytes loaded with fluorescent dye Alexa Fluor 594 through a patch pipette in human cortical slices from patients of both sexes. The z-stacks of images acquired with two-photon microscopy were used for three-dimensional (3D) reconstructions of individual astrocytes (Fig. 2a). Subsequently, 3D Sholl analysis was performed on these reconstructions (Fig. 2b). We observed a decrease in astrocytic size and complexity in older human adults. Both the branch length and the maximal number of intersections decreased (Fig. 2c, d and Supplementary Fig. 4a, b). However, neither the number of primary branches nor the ramification index was significantly affected (Fig. 2e, f and Supplementary Fig. 4c, d).

Then we estimated the sizes of astrocyte territorial domains by measuring the area of astrocyte image projections along the z-axis (Fig. 2g). Consistent with reduced branch length, the astrocyte domains were smaller in older adults (Fig. 2h and Supplementary Fig. 4e). Smaller domains may contribute to an increase in extracellular space and uncoupling of astrocytes^9,14^. Therefore, we counted the numbers of coupled astrocytes stained by fluorescent dye diffusion from the patched cell and found that the number of coupled astrocytes was smaller in older adults (Fig. 2i, j and Supplementary Fig. 4f).

Protoplasmic astrocytes in the human brain contact up to 2 million synapses. These contacts are mainly formed by tiny astrocytic processes known as leaflets^1^. Since astrocytic leaflets are beyond the optical resolution of the two-photon microscopy, we estimated leaflet volume fraction (VF). This method assumes fluorescence measured from astrocytic soma corresponds to 100% space occupancy. Hence, the fluorescence of unresolved astrocytic leaflets normalized to the fluorescence of soma yields the VF of the leaflets^12,15^. The leaflet VF was significantly lower in older adults, indicating decreased leaflet size, density, or both (Fig. 2k and Supplementary Fig. 4g).

Shrinkage of astrocytes and loss of gap junctional coupling can affect the electrical properties of these cells^9,14^. In the whole-cell voltage-clamp mode, we recorded astrocytic currents in response to depolarization steps and plotted current–voltage (IV) relationships (Fig. 2l). Shallower slop of the IV relationships in older adults indicates higher cell input resistance (Fig. 2m and Supplementary Fig. 4h).

### Astrocytic atrophy is associated with increased GFAP and decreased ezrin expression

**Fig. 3:**
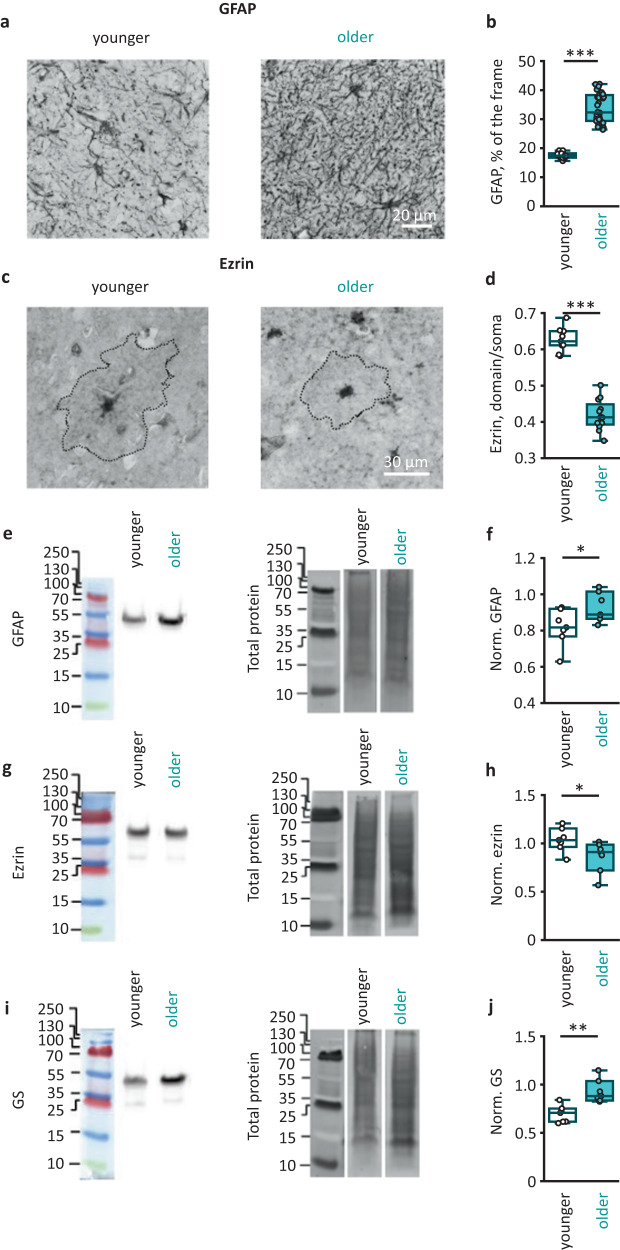
Age-dependent GFAP and GS upregulation and ezrin deficiency. **a** Representative examples of immunostaining for glial fibrillary acidic protein (GFAP) in cortical tissue from younger (left) and older (right) adults. **b** Percentage of the image covered by pixels stained for GFAP in two age groups (*P* < 0.001; younger adults: *N* = 3 people, *n* = 24 images; older adults; *N* = 4 people, *n* = 34 images). **c** Representative examples of immunostaining for ezrin in cortical tissue from younger (left) and older (right) adults. The astrocytic territorial domains are outlined by dotted line. **d** Ezrin immunostaining intensity averaged in the astrocyte territorial domain and normalized to the immunostaining intensity of soma in two age groups (*P* < 0.001; younger adults: *N* = 3 people, *n* = 9 cells; older adults: *N* = 4 people, *n* = 12 cells). **e** Representative Western blots of cortex homogenates stained by antibodies against GFAP (left) and total protein bands (right). **f** GFAP amount normalized to total protein amount in two age groups (*P* = 0.04; younger adults: *N* = 7 people; older adults: *N* = 7 people; *n* = *N*). **g**, **h** Same as (**e**, **f**) but for ezrin (*P* = 0.005; *N/n*—numbers are the same as for (**f**)). **i**, **j** Same as (**e**, **f**) but for glutamine synthetase, GS (*P* = 0.03; *N/n*—numbers are the same as for (**f**)). Data are shown as box-and-whisker plots where the box is Q1 and Q3 with median, and whiskers are the ranges within 1.5IQR. Empty boxes/circles—younger adults, filled boxes/circles—older adults. Two-tailed (except for (**f**), where one-tailed) Mann–Whitney test: ^N.S.^*P* > 0.05, *P < 0.05, **P < 0.01, ***P < 0.001. Source data are provided as a Source Data file.

Age-dependent upregulation of GFAP is well documented^16,17^. Based on increased GFAP immunostaining, it was concluded that astrocytes become reactive in old age^18,19^. Here, we also observed increased GFAP immunostaining in the cortex of older adults (Fig. 3a, b and Supplementary Fig. 5a). This increase in GFAP immunoreactivity was not accompanied by a significant change in the number of GFAP-positive astrocytes in older adults, suggesting an increase in the expression of this protein within the cells (Supplementary Fig. 6). Increased GFAP expression in astrocytic soma and proximal branches does not contradict observed astrocyte atrophy manifested in shrinkage of branches and leaflets that do not contain GFAP^9^. Consistent with a previous report^20^, GFAP expression negatively correlated with immunostaining for membrane-to-actin cytoskeleton linker ezrin essential for the formation and plasticity of astrocytic leaflets (Fig. 3c, d and Supplementary Fig. 5b). These results were supported by Western blotting of human brain samples. In older adults, the amount of GFAP protein in the cortical tissue significantly increased (Fig. 3e, f and Supplementary Fig. 5c), whereas the amount of ezrin decreased (Fig. 3g, h and Supplementary Fig. 5d). We also observed a significant increase in the protein level of glutamine synthetase (GS) in the older tissues (Fig. 3i, j and Supplementary Fig. 5e).

### Neuronal electrical properties and sIPSCs are not altered in the aged neocortex

**Fig. 4:**
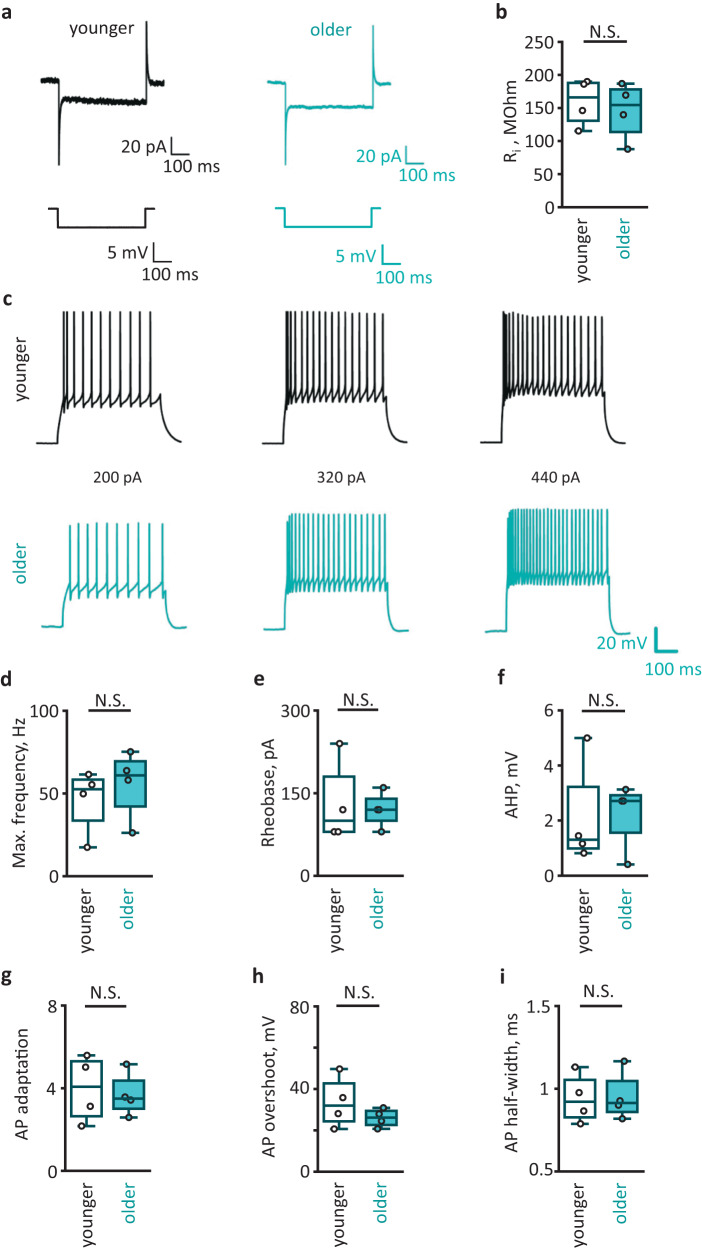
Aging does not affect membrane properties and excitability of pyramidal neurons. **a** Representative currents recorded in cortical pyramidal neurons in response to 5 mV voltage steps in younger (left) and older (right) adults. **b** Input resistance (R_i_) of neurons in two age groups (*P* = 0.66; younger adults: *N* = 4 people; older adults: *N* = 4 people; cell number *n* = *N*). **c** Action potentials (APs) recorded in current-clamp mode in response to current steps 200 pA, 320 pA, and 440 pA in younger (top) and older (bottom) adults. **d** Maximal frequency of Aps (*P* = 0.3); **e** rheobase (*P* = 0.9); **f** afterhyperpolarization (AHP, *P* = 0.99); **g** AP adaptation (*P* = 0.5); **h** AP overshoot (*P* = 0.9); **i** AP half-width in younger and older adults (*N/n*—numbers the same as for (**b**)). Data are shown as box-and-whisker plots where the box is Q1 and Q3 with median, and whiskers are the ranges within 1.5IQR. Empty boxes/circles—younger adults, filled boxes/circles—older adults. Two-tailed Mann–Whitney test: ^N.S.^*P* > 0.05. Source data are provided as a Source Data file.

We performed whole-cell patch-clamp recordings from layer III - IV pyramidal neurons in two age groups. Firstly, we injected hyperpolarizing voltage steps and measured the input resistance of the cells in voltage-clamp mode (Fig. 4a). No significant change in the input resistance was observed in aged neurons (Fig. 4b). Then, we injected depolarizing current steps with increasing amplitude and recorded action potentials (APs) in current-clamp mode (Fig. 4c). We did not observe significant differences for any measured parameters: maximal frequency, rheobase, afterhyperpolarization (AHP), AP adaptation, AP overshoot, AP half-width between two age groups (Fig. 4d–i). These results indicate that the electrical properties of the neuronal membrane, including its excitability, do not change in the aged neocortex.

This result does not exclude that aging affects synaptic transmission. Impairment of synaptic transmission could logically follow reduced synaptic coverage due to astrocyte atrophy. In our experimental settings, we could monitor spontaneous inhibitory postsynaptic currents (sIPSCs) in voltage-clamp mode. However, we did not observe significant changes in sIPSC amplitude, decay, or frequency (Supplementary Fig. 7).

## Discussion

Single-cell Raman spectrum analysis revealed that aging is associated with mitochondrial malfunction in astrocytes but not in neurons. In particular, we found that the levels of reduced *c- b* and *a*-types cytochromes were lower in aged astrocytes, reflecting the decreased amount of electrons in ETC. Fewer electrons in ETC may reflect either a higher rate of electron transfer or an overall decrease in the ETC supply with the primary electron donors (or both). If the supply of electron donors drops, we should see a proportional decrease in the amount of different types of reduced cytochromes. Instead, we observed a more pronounced decline in the relative amount of reduced *c*-type cytochromes than in the relative amount of reduced *b*-type cytochromes in astrocytes. This result is consistent with faster (more efficient) electron transfer from *c*-type cytochrome to complex IV. In agreement with this finding, we observed an age-dependent decrease in the relative amount of reduced *a*-type cytochromes in the complex IV in astrocytes.

A faster electron transfer rate may correlate with decreased reactive oxygen species (ROS) production in astrocytes in old adults. The formation of O_2_^-^ occurs because of the reversed electron transport from ubiquinol to complex I following a slowdown of the electron transport in the downstream ETC part, particularly in complex IV or between c-type cytochrome and complex IV leading to the ETC overload with electrons^21^. A higher transfer rate from *c*-type cytochrome to complex IV should reduce ubiquinol-mediated reversed transport. Moreover, our results suggest a lower probability of the electron transfer to O_2_^−^ in complex III in the semiquinone-binding site.

A decrease in reactive oxygen species (ROS) production in astrocytes of older adults most likely has consequences for brain function in general. Although excessive ROS concentrations are the signature of oxidative stress, their physiological concentrations are important for healthy brain function^22^.

In contrast to decreased ROS production, a faster electron transfer rate in ETC does not necessarily affect oxidative phosphorylation. Oxidative phosphorylation depends on the number of transferred electrons and, hence, the amount of electron donors. How aging affects electron donors and mitochondrial ATP production in astrocytes remains to be determined. ATP is critical for astrocytic homeostatic function, as ATP fuels Na^+^/K^+^ ATPase responsible for K^+^ buffering and maintenance of Na^+^ gradients sustaining homeostatic transporters^23–25^. Reduced ATP supply may affect critical astrocytic support of neuronal excitability and neurotransmission.

Aging leads to morphological atrophy of cortical protoplasmic astrocytes: the length of astrocytic branches decreases, and the territorial astrocytic domain shrinks. This observation may explain an increase in extracellular space with age^9,26^. Astrocytic morphological changes are fundamental for the regulation of extracellular space^27^. An increase in extracellular space can also affect the extracellular diffusion of neurotransmitters, neuromodulators, and ions, as well as the operation of the glymphatic system^28^. Aging is associated with a substantial reduction of the VF of astrocytic leaflets. Age-related decrease in VF can reflect a lower number of leaflets, smaller sizes, or both. The precise mechanism requires direct measuring of leaflet sizes and numbers. Smaller leaflet density can occur because reduced branches surface is available to carry the leaflets in older adults. Nevertheless, the lower density of leaflets indicates a reduction of astrocytic interaction with other components of the brain’s active milieu, including synapses^2^. Synapses in the old brain therefore, have less astrocyte associations, which may limit homeostatic support, facilitate the spillover of neurotransmitters, and affect synaptic plasticity^29^.

Along with astrocytic morphological atrophy, we observed increased astrocyte input resistance. This increase most likely reflects a decrease in the membrane surface of the astrocytes, translating into a lower total membrane conductance. In addition, shrinkage of astrocytes was associated with the uncoupling of astrocytes through gap junctions. Similar uncoupling was observed in aged mice^14^. Loss of gap junction can also increase cell input resistances, and compromise astrocyte isopotentiality within syncytium^30^. Such age-dependent changes in electric properties can significantly affect astrocyte function. For example, potassium released during synaptic transmission depolarizes perisynaptic astrocytic processes and suppresses voltage-dependent glutamate uptake^31^. Cell input resistance defines membrane time and length constants, thus modulating the temporal and spatial properties of the glutamate uptake in the vicinity of active synapses. An increase in astrocyte input resistance prolongs and spreads astrocytic depolarization, reducing glutamate uptake in old age.

Astrocytic atrophy was associated with an increase in the expression of GFAP. Morphological analysis based on GFAP immunostaining is generally accepted as a sign of astrocytic hypertrophy and reactivity^32^. On the other hand, GFAP immunoreactivity is not detected in all astrocytic branches and is absent in leaflets^1^. Using astrocyte staining with a fluorescent dye, we revealed entire cell morphology and observed morphological atrophy in older adults. Moreover, GFAP upregulation can trigger a deficiency of ezrin^20^. Since ezrin is essential for the formation of leaflets, the downregulation of this protein may be responsible for their loss or reduction in size. Furthermore, the upregulation of GFAP can be linked to age-related astrocyte uncoupling, which is also detrimental to learning and memory^33^. Aging was also associated with a significant increase in astrocytic expression of GS that converts glutamate and ammonium to glutamine. Upregulation of GS may serve to reduce the toxicity of ammonia, levels of which increase in the aged brain^34^. It may also signal some adaptive changes in the glutamate (GABA) glutamine shuttle^4^.

The mitochondrial malfunction in aged astrocytes was not accompanied by similar changes in neurons. Raman microspectroscopy did not reveal changes in electron loading of neuronal ETC. It may reflect either a lack of metabolic changes or reduced activity of the Krebs cycle because of reduced energy demand and ATP production. Nonetheless, unchanged ETC loading with electrons does suggest the absence of changes in O_2_^−^ generation. It also indicates the absence of mitochondrial damage or some severe ETC malfunctions in neurons.

Moreover, we observed a decrease in the protein-to-lipids ratio in astrocytes and an increase in neurons. Accumulating misfolded and damaged proteins in the brain were proposed as a determinant of brain aging^35^. Nonetheless, no significant changes in basic excitability and membrane properties were observed in the neurons of older adults. No significant changes in sIPSC amplitude, decay, and frequency were observed either. Thus, impaired protein clearance may not immediately affect neuronal function. The relatively preserved neuronal function does not exclude that astrocytic atrophy can affect synaptic transmission and plasticity during physiological brain activity. The diminished presence of astrocytic leaflets in the brain’s active milieu is likely to compromise neurotransmitter uptake and potassium clearance^14^. Extracellular accumulation of potassium could affect neuronal excitability, presynaptic release of glutamate, and its uptake by astrocytes^31,36^.

One limitation of this study is that the results were obtained in the cortical layer 2/3 and different parts of the neocortex. We cannot exclude heterogeneity of aging in different parts of the cortex and subcortical regions. Moreover, the brain’s active milieu consists not only of astrocytes and neurons. Other cell types and non-cellular elements may also be affected by aging. Microglial reactivity and prominent microglial atrophy were reported in the aging brain^37,38^. The structure and mechanical properties of the extracellular matrix also undergo significant alterations^39^. Vascular aging, malfunctional blood–brain barrier, and impaired angiogenesis can contribute to cognitive decline^40–43^. Since all these components interact with each other, the malfunction of one affects the others. Hence, further studies comparing the timecourses of age-related changes in different elements of the brain-active milieu and causal relationships between these changes are needed.

Another salient limitation of the current study is that cortical tissue was obtained from patients undergoing resection of gliomas or other cancer metastases to the brain. It is well established that tumors can significantly affect different cell types in the surrounding brain-active milieu: neurons, astrocytes, microglia, blood vessels, etc.^44–46^. Although we always tried to collect neocortical tissue from patients with tumors located in the deep subcortical regions, we could not entirely exclude the possibility that the illness and treatment may affect the recorded parameters while aging affects the healthy brain in a different way.

In conclusion, we found that brain aging is associated with atrophy and mitochondrial malfunction of cortical astrocytes, but not neurons. Moreover, neurons preserve their excitability in older adults. These findings do not rule out other age-related changes in neurons. For example, the protein-to-lipid ratio was decreased in astrocytes and increased in neurons during aging. How the latter affects neuronal function has yet to be determined.

## Methods

### Human cortical slices

The specimens of neocortical tissue were obtained during tumor resection from 42 patients of both sexes in the range of ages from 18 to 71 years. A surgical approach to the tumor was performed using a frameless navigation system with uploaded functional MRI data and intraoperative neurophysiological monitoring (as well “awake” surgery) for the preservation of motor and speech eloquent brain areas and white matter tracts^47^. During the resection, a fragment of neocortical tissue was removed to get access to the tumor. The tissue was collected from the access area outside the area of changes on the T2-FLAIR MRI. Neocortical tissue was put in a cutting solution containing (in mM): 85 NaCl; 2.5 KCl; 26 NaHCO_3_ 1 NaH_2_PO_4_; 7 MgCl_2_; 0.5 CaCl_2_ and 50 sucrose. The solution had an osmolarity of 295 ± 5 mOsm and pH of 7.4 when saturated with 95% O_2_ and 5% CO_2_. The cortical region varied according to the surgery protocol and location of the tumor. The study was always performed on the cortical layer 2/3. The study was approved by the Ethical Committee of the Privolzhsky Federal Research Medical Centre of the Ministry of Health of the Russian Federation, and informed patient consent was obtained.

Neocortical tissue was cut into 350 µm slices with a vibrating microtome HM650 V (Thermo Fisher Scientific, Waltham, USA) in the cutting solution. After preparation, the slices were incubated for recovery for 1 h at 32–34 °C in a storage solution containing (in mM): 92 NaCl; 2.5 KCl; 30 NaHCO_3_ 1.2 NaH_2_PO_4_; 1 MgCl_2_; 1 CaCl_2_; 5 Na-ascorbate; 3 Na-pyruvate; 2 HEPES; 2 Thiourea and 25 d-glucose. The solution had an osmolarity of 295 ± 5 mOsm and pH of 7.4 and was saturated with carbogen (95% O_2_ and 5% CO_2_). For electrophysiology recordings and two-photon imaging, the slices were placed in an immersion chamber where they were continuously superfused (1–3 ml/min) with constantly carbogenized solution containing (in mM): 127 NaCl; 2.5 KCl; 1.25 NaH_2_PO_4_; 1 MgCl_2_; 2 CaCl_2_; 25 NaHCO_3_ and 25 D‐glucose. The solution had an osmolarity of 295 ± 5 mOsm, pH of 7.4, and temperature of 34 °C. The cells were selected at a depth of about 50 µm to avoid damaged cells on the surface but still obtain good fluorescent images.

### Raman microspectroscopy

Cortical slices were immunocytochemically double stained for GFAP and NeuN to identify, respectively, astrocytes and neurons for subsequent Raman microspectroscopy. The staining was performed in the following steps: (1) acute slices after the recovery period were placed into 4% paraformaldehyde (PFA) solution (37 °C) for 60 min and then washed twice in phosphate buffer solution (PBS, pH 7.4); (2) PFA-fixed slices were transferred into PBS with 0.3% Triton-X100 solution for 15 min and then into PBS with 0.1% Tween-20 and 5% BSA solution (25 °C) for 90 min; (3) the slices were incubated in the solution of primary antibodies: anti-glial fibrillar acidic protein (GFAP) rabbit polyclonal antibody (1:1000, Abcam, Cambridge, UK, catalog number ab7260) and anti-neuronal nuclear protein (NeuN) chicken polyclonal antibody (1:50, Novus Biologicals, Centeennial, USA, catalog number NBP2-10491) in PBS with 0.01% Tween-20 for 60 h at 25 °C; (4) the slices were washed three times in PBS, 10 min, at 25 °C; (5) the slices were incubated in the solution of secondary antibodies: Cy2 AffiniPure donkey anti-rabbit IgG (H + L) (1:200, Jackson ImmunoResearch, Ely, UK, catalog number 711-225-152) and Cy5 AffiniPure donkey anti-chicken IgG (H + L) (1:200, Jackson ImmunoResearch, catalog number 703-175-155) for 2 h at 25 °C; (5) the slices were washed twice in PBS and in trice in deionized water. Then, the slices were placed on the glass slide without covering with the cover glass and dried in the dark. Stained slices were stored at room temperature in the dark. The secondary antibodies were chosen according to the spectral properties of their fluorophores (excitation and emission wavelengths, Cy2: *λ*_ex_ = 492 nm; *λ*_em_ = 510 nm and Cy5: *λ*_ex_ = 650 nm; *λ*_em_ = 670 nm) so they did not emit fluorescence upon 532 nm laser excitation used for Raman spectroscopy.

Raman spectra were recorded with confocal Raman microspectrometer NTEGRA SPECTRA (NT-MDT, Zelenograd, Russia) attached to the inverted Olympus microscope. The brain slices were placed on the glass slides without a cover glass. The samples were illuminated by the laser from the side of the slice to avoid contamination of Raman spectra by fluorescence emitted by the glass. Astrocytes and neurons were identified by Cy2 and Cy5 fluorescence, respectively. Then, Raman spectra were recorded from the somas of the identified astrocytes and neurons with the ×40 NA 0.45 objective following laser excitation at 532 nm (1 mW power per registration spot with the diameter of ~800 nm and *z*-thickness of 1.5 µm) and from astrocytic soma with the same objective and laser excitation at 633 nm (3 mW). The spectrum accumulation time was 60 s for both lasers. The laser excitation at 532 nm produced negligible Cy2 or Cy5 fluorescence without interfering with the Raman scattering of the stained cells. The total number of identified cells studied with 532 nm laser was 60 astrocytes (7 patients) and 58 neurons (7 patients). The total number of astrocytes studied with 633 nm laser was 34 (7 patients).

Raman spectra were analyzed with the open-source software Pyraman, available at https://github.com/abrazhe/pyraman. The baseline was subtracted in each spectrum. The baseline was defined as a cubic spline interpolation of a set of knots, numbers, and x-coordinates, which were selected manually outside any informative peaks in the spectra. Once chosen, the number and *x*-coordinates of the knots were fixed for all spectra in the study. *Y*-coordinates of the knots were defined separately for each spectrum as 5-point neighborhood averages of spectrum intensities around the user-specified *x*-position of the knot. The parameters for baseline subtraction were chosen after processing ~50 spectra from different astrocytes and neurons.

Analysis of Raman spectra obtained with 532 nm laser excitation: After the baseline subtraction, the intensities of peaks with the following maximum positions were defined: 750, 1126, 1440, and 1660 cm^−1^. Peaks at 750 and 1126 cm^−1^ correspond to bond vibrations in hemes of reduced cytochromes of *c*- and *b*-types, with the main contribution from *c*-type cytochromes for the first peak and the main contribution from *b*-type cytochromes for the second peak^48–50^. Peaks at 1440 and 1660 cm^−1^ correspond to vibrations of C-C bonds in lipids and peptide bonds in proteins, respectively^48^. Relative Raman peak intensities were used to obtain the protein amount normalized to the lipid amount (*I*_1660_/*I*_1440_ ratio), the amount of reduced *c*- and *b*-type cytochromes (with the main contribution from reduced c-types cytochromes) normalized on the protein amount (*I*_750_/*I*_1660_ ratio), the ratio of reduced *c*-type cytochromes to reduced *b*-type cytochromes (*I*_750_/*I*_1126_ ratio) in astrocytes and neurons^48^.

Analysis of Raman spectra obtained with 633 nm laser: Raman spectra obtained with 633 nm excitation have the same “lipid” and “protein” peaks with maximum positions at 1440 and 1660 cm^−1^, as in spectra with 532 nm excitation, and the broad peak in the region 1550–1580 cm^−1^ corresponding to vibrations of different heme bonds in reduced a-type cytochromes^51^. To estimate the relative amount of reduced a-type cytochromes, we used the ratio of the sum of intensities in the regions 1550–1580 cm^−1^ and 1655–1665 cm^−1^ (*I*_[1550-1580]_/*I*_[1655-I1665]_).

### Electrophysiological recordings

Astrocytes were selected at the border of cortical layers II–III. Whole-cell recordings were performed with borosilicate glass pipettes (5–7 MΩ) filled with an internal solution containing (in mM): 135 KCH_3_SO_3_, 10 HEPES, 10 Na2phosphocreatine, 8 NaCl, 4 Na2ATP and 0.4 NaGTP (pH was adjusted to 7.2 with KOH; osmolarity to 290 mOsm). 50 μM Alexa Fluor 594 (Thermo Fisher Scientific, USA) was added to the solution to reveal cell morphology. Passive astrocytes were identified by strongly negative resting membrane potential and linear current–voltage (IV) relationship. In the current-clamp mode, current steps were applied to corroborate the absence of membrane excitability. In voltage-clamp recordings, the astrocytes were held at −80 mV. Voltage steps (Δ20 mV; 500 ms) from −140 to +80 mV were applied to obtain IV relationships. All responses were amplified with a Multiclamp 700B amplifier (Molecular Devices, San Jose, USA), digitized with digital-analog converter board NI PCI-6223 (National Instruments, Austin, USA), and recorded with WinWCP v5.2.5 software (University of Strathclyde, UK). The data were analyzed with the Clampfit 10.4 software (Molecular Devices, USA).

Neurons were selected in cortical layer III. Whole-cell recordings were performed with borosilicate glass pipettes (4–5 MΩ). Cell excitability was measured with a pipette solution containing (in mM): 140 K-gluconate, 8 NaCl, 0.2 CaCl_2_, 10 HEPES, 2 EGTA, 0.5 NaGTP, and 2 MgATP (pH was adjusted to 7.2 with KOH and osmolarity to 290 mOsm). Membrane potential was manually set at −70 mV in current-clamp mode. We recorded cell spiking in response to 500-ms current steps of increasing amplitudes from 0 to 440 pA in steps of 40 pA. The maximum frequency of action potentials was calculated in response to the current step of 440 pA. The rheobase was calculated as the amplitude of the previous current step, after which action potentials appeared. The afterhyperpolarization (AHP) was calculated as the amplitude of a negative peak appearing after the depolarizing step relative to the baseline before the depolarizing step. We calculated the spike adaptation rate as a ratio of the instantaneous frequency of the first and the second AP to the instantaneous frequency of the last and previous AP in response to 440 pA current step. AP overshoot was calculated as AP peak value over 0 mV. AP half-width was calculated as the duration of the action potential at the voltage halfway between the threshold and the action potential peak. Cell input resistance was calculated in voltage-clamp mode from the current in response to 5 mV depolarizing step.

GABA_A_ receptor-mediated sIPSCs were recorded in voltage-clamp mode (holding potential −70 mV) in the presence of 25 μM NBQX, 50 μM APV, and 5 μM CGP52432 to block AMPA, NMDA, and GABA_B_ receptors, respectively. The recordings were done with intracellular solution containing (in mM): 135 CsCl, 10 HEPES, 10 phosphocreatine, 4 MgATP, 0.3 TrisGTP, 0.3 EGTA (pH 7.35 was adjusted with CsOH). sIPSCs were identified using a semi-automated detection software Mini Analysis 6.0.7 (Synaptosoft, Fort Lee, NJ, USA). The amplitude, frequency, and decay time constant of sIPSCs were measured. sIPSC amplitude and decay were averaged in individual sweeps.

### Astrocyte morphological analysis

One randomly selected astrocyte per patient was used for morphological analysis. Cells were filled with 50 µM Alexa 594 through a patch pipette. Then, we waited at least 10 min to allow for sufficient dye diffusion in the astroglial syncytium. Astrocyte imaging was performed with a two-photon laser scanning microscope Zeiss LSM 7 MP (Carl Zeiss, Germany) equipped with femtosecond laser Chameleon Vision II (Coherent, UK). Alexa 594 was excited at 830 nm. *Z*-stacks of 100 images (512 × 512 pixels size, 0.2 µm/px) were collected with 1 µm steps. *Z*-axis increments were re-sampled by spline interpolation to match the lateral resolution of 0.2 µm/px. For astrocytes morphometry, we used Image-funcut (image-funcut, https://github.com/abrazhe/image-funcut), Scikit-Image (scikit, http://scikit-image.org/), and Sci-Py (scipy, http://www.scipy.org/) libraries^52,53^.

Before analysis, coherence-enhancing diffusion filtering^54^ was applied to enhance the contrast of anisotropic structures, such as astrocytic processes. After the filtering, the astrocytic domain was segmented by applying a hysteresis threshold (high threshold: 3× standard deviations of brightness, low threshold: 1× standard deviation of brightness levels) within each *Z*-plane, removing contiguous structures smaller than 9 pixels. Finally, the masks for each *Z*-plane were combined, and contiguous structures with less than 100 voxels were again removed. The resulting three-dimensional mask was used for visualization in Fig. 2a and, after max-projection along the *Z*-axis to estimate domain area (Fig. 2g, h).

Astrocytic branches were traced with the Simple neurite tracer plugin (https://github.com/morphonets/SNT) for ImageJ software version 1.53 (NIH, Bethesda, USA)^55^. Then, 3D Sholl analysis was performed: the center of the soma was taken as the center for the set of concentric spheres with increasing radius (from 10 to 100 µm with a step of 1 µm). We collected the numbers of individual astrocytic branches intersected by each sphere to build the Sholl profile: a graph of the number of intersections versus the distance from the center of the soma. The number of primary branches, the maximal number of intersections, and the ramification index (the ratio of maximal intersection to primary branches) were calculated from the Sholl analysis. In addition, we calculated the mean branch length and the astrocyte domain area (the area circumscribed by a line connecting endpoints of the traced astrocyte processes in astrocyte maximal projection).

Next, we chose the frame within the *Z*-stack containing astrocyte soma. Special attention was paid that the fluorescence of soma was not saturated^56^. We constructed five fluorescence cross-sections passing through the center of the soma (72° from each other). We discarded large fluctuations (>10% and >0.5 µm) of fluorescence corresponding to astrocytic branches. Next, we estimated the volume fraction (VF) of astrocytic leaflets as relative fluorescence intensity in the unresolved processes normalized to fluorescence intensity in the soma. Characteristic VF was obtained at a distance of 30–40 µm from the soma boundary.

### Immunocytochemical analysis

The cortical slices were fixed in zinc-ethanol-formaldehyde^57^, dehydrated, and embedded in paraffin. Paraffin blocks were cut into 5-μm sections. After the standard dewaxing procedure, the sections were unmasked by heating in modified citrate buffer S1700 (Dako, Glostrup, Denmark). Then, the activity of endogenous peroxidase was blocked in 3% hydrogen peroxide solution for 10 min. Then the tissue was blocked in Protein Block solution (Spring Bioscience, Pleasanton, USA) for 10 min. The sections were incubated for 48 h in monoclonal (clone 3C12) mouse anti-ezrin antibodies (1:100, Diagnostic BioSystems, Pleasanton, USA, catalog number Mob380) in monoclonal (clone GA5) mouse anti-GFAP antibodies (1:100, Biocare Medical, Pacheco, USA, catalog number CM065). Reveal Compliment and Reveal HRP-conjugated solution from The Reveal Polyvalent HRP DAB Detection System kit (Spring Bioscience, USA) was used as a secondary reagent. The secondary antibody was applied for 30 min, according to the manufacturer’s instructions. Ezrin was visualized using 3,3’-diaminobenzidine from the DAB+ kit (Agilent, Santa Clara, USA). Then, all diaminobenzidine-developed samples for brightfield microscopy were mounted with the permanent medium Cytoseal 60 (Thermo Fisher Scientific). Samples were imaged with Leica DM750 microscope and an ICC50 camera (Leica, Germany). The mean intensity of immunostaining of ezrin in the astrocyte territorial domain was normalized to the mean intensity of immunostaining of soma. The percentage of pixels with GFAP immunostaining exceeding a threshold (>10% of soma intensity) was calculated in each frame.

### Western blotting

Cortical tissue from the patients was frozen in liquid nitrogen. Then each tissue block was homogenized in the RIPA buffer containing SIGMAFAST protease inhibitor cocktail (Sigma-Aldrich, St. Louis, USA), diluted in the loading buffer (120 mM Tris-HCl, 20% [v/v] glycerol, 10% [v/v] mercaptoethanol, 4% [w/v] sodium dodecyl sulfate, and 0.05% [w/v] bromophenol blue, pH 6.8), loaded to SDS-PAGE, and blotted onto nitrocellulose membranes (GE Healthcare, Chicago, USA). The membranes were blocked in 5% skim milk (Sigma-Aldrich) in the TBS buffer (50 mM Tris, 150 mM NaCl, pH 7.4) + 0.2% Tween-20 (Applichem, Darmstadt, Germany) for 1 h at room temperature and then incubated overnight at 4 °C either with primary mouse antibodies for ezrin (1:1000; Antibodies-Online, clone 6F1A9, catalog number ABIN5542456, Lot number 141031, Aachen, Germany), or with primary rabbit antibodies for GFAP (1:1000; Antibodies-Online, catalog number ABIN3044350, Lot number 0901412Da248285), or with primary guinea pig antibodies for GS (1:1000, Synaptic Systems, Catalog number 367 005, Lot number 367005/6, Goettingen, Germany). After incubation with primary antibodies, membranes were rinsed in TBS with 0.2% Tween-20 and incubated with corresponding HRP-conjugated secondary antibodies: either with anti-mouse IgG (1:5000, Jackson Immunoresearch, West Grove, USA, catalog number 715-035-150, Lot number 158318), or anti-rabbit IgG (1:5000, Abcam, catalog number 6721, Lot number GR231489-1), or anti-guinea pig IgG (1:5000, Jackson Immunoresearch, catalog number 706-035-048, Lot number 144939) for 1 h. ECL substrate (Bio-Rad, Hercules, USA) was used for signal detection. Protein bands were visualized using the ImageQuant LAS500 chemidocumenter (GE Healthcare, Chicago, USA). The ECL signal was visualized in the chemiluminescent channel, and the protein marker was detected in the optical channel of the chemidocumenter. The intensity of the protein bands was quantified using the gel analyzer option of ImageJ software version 1.53 (NIH, Bethesda, USA). To exclude inter-sample variability, the averaged intensities of the bands measured for each patient/protein were normalized to the average intensity of the lanes with the total protein of the corresponding samples using the No-Stain labeling kit (A44449, Lot number 2365205, Life Technologies, Carlsbad, USA) using the fluorescent channel of the chemidocumenter.

### Statistics and reproducibility

Several cortical slices were collected from each patient. In patch-clamp recordings, only one cell was recorded per slice. N-number indicated the number of people in the sample, and n-number indicated the number of slices, cells, or samples used. Because of ethical considerations, no statistical method was used to determine the sample size. The sample size was determined by the availability of the tissue. No assumption was made about the data distribution. Hence non-parametric Mann–Whitney statistical test was used. *P* > 0.05 considered non-significant (N.S.) The following values were considered significant *P  <  0.05, **P < 0.01, ***P < 0.005. All data are presented as box-and-whisker plots. Whiskers show 1.5IQR. No data were excluded from the analyses, the experimenters were not blinded.

### Reporting summary

Further information on research design is available in the Nature Portfolio Reporting Summary linked to this article.

### Supplementary information

Supplementary Information

Reporting Summary

### Source data

Source Data

## Data Availability

The raw data generated in this study are protected and unavailable due to data privacy law (the Russian Federal Law on Personal Data No. 152-FZ). The processed data are included in the manuscript and supporting files in aggregated form. Source data are provided with this paper.

## Code availability

For astrocyte morphometry, we used Image-funcut (10.5281/zenodo.10151748) 10.5281/zenodo.10151748. Raman spectra were analyzed with the open-source software Pyraman (10.5281/zenodo.10151752) 10.5281/zenodo.10151752.
