# Supplementary material for: Mitochondrial malfunction and atrophy of astrocytes in the aged human cerebral cortex
Source: Nat Commun. 2023 Dec 16;14:8380. doi: 10.1038/s41467-023-44192-0 (PMC10725430; doi:10.1038/s41467-023-44192-0)
Supplement: Supplementary file 1 — Supplementary Information [file 41467_2023_44192_MOESM1_ESM.pdf]

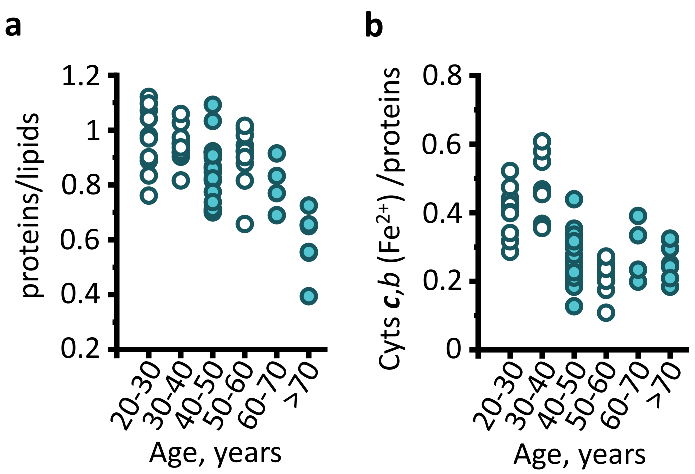

Supplementary Figure 1. Age- and sex-dependency of metabolic changes in human astrocytes.

a. The ratio of proteins to lipids. **b.** The relative amount of reduced cytochromes of *c*,*b*-types. Data obtained from individual cells (n = 58) of 7 patients at different ages. Empty circles are females, filled circles are males.

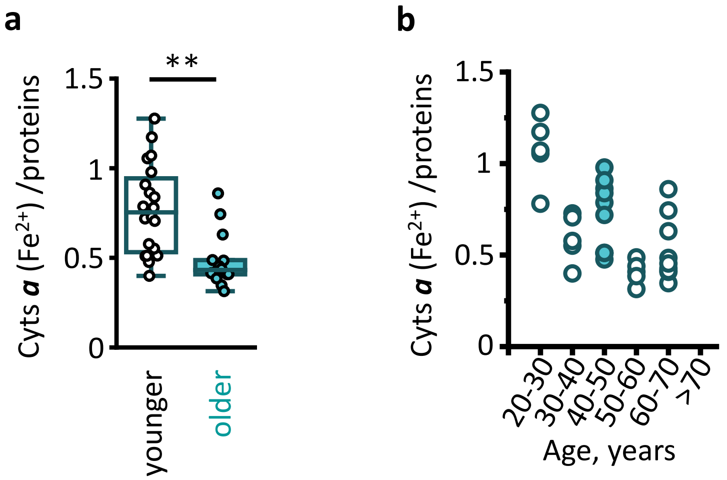

Supplementary Figure 2. Age- and sex-dependency of changes in astrocytic *a*-type cytochromes.

The relative amount of reduced cytochromes of *a*-types in two age groups (**a**) and in individual patients at different ages (**b**).
Data obtained from individual cells (n = 34) of 7 patients at different ages. Empty circles are females, filled circles are males. Mann-Whitney test: ** – $p < 0.01$.

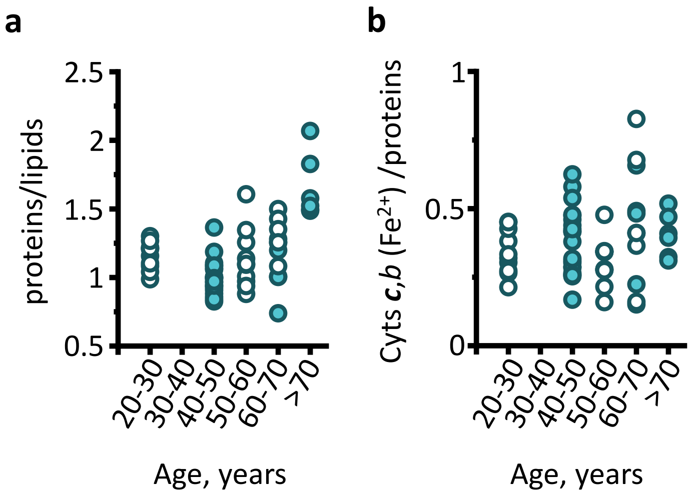

Supplementary Figure 3. Age- and sex-dependency of metabolic changes in human neurons.

a. The ratio of proteins to lipids. **b.** The relative amount of reduced cytochromes of *c*,*b*-types. Data obtained from individual cells (n = 58) of 7 patients at different ages. Empty circles are females, filled circles are males.

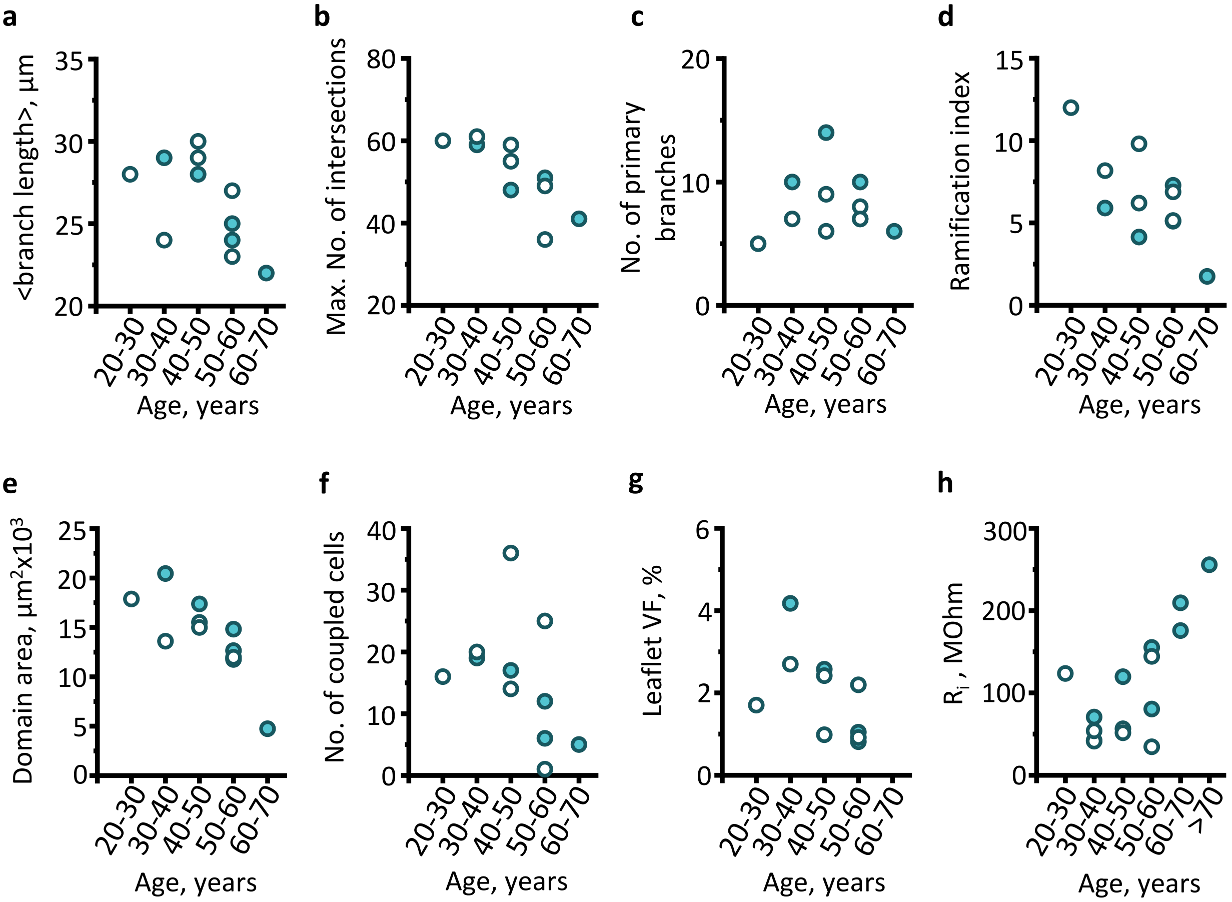

Supplementary Figure 4. Age- and sex-dependency of astrocytic atrophy.

The results of 3D Sholl analysis of cortical astrocytes in 11 patients at different ages: **a.** mean branch lengths, **b**. maximum numbers of intersections, **c**. numbers of primary branches, and **d**. ramification indexes. **e.** Astrocytic domain areas in 11 patients at different ages. **f.** Numbers of coupled astrocytes in 11 patients at different ages. **g.** Volume fractions (VF) of astrocytic leaflets in 11 patients at different ages. **h.** Input resistances (R_i) of astrocytes in 13 patients (15 cells) at different ages.

Data obtained from individual cells. One cell per patient, except for panel h. Empty circles are females, filled circles are males.

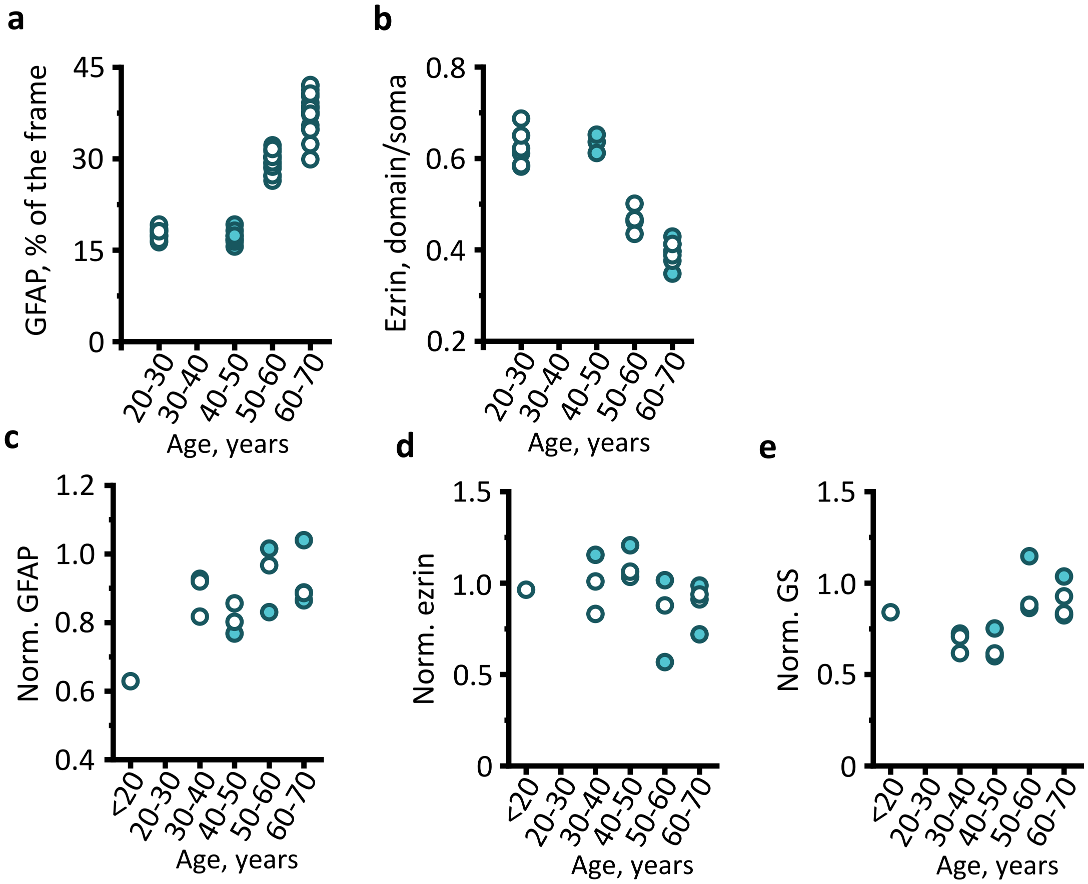

Supplementary Figure 5. Age- and sex-dependency of GFAP and GS upregulation, and Ezrin deficiency.

a. Percentage of the image covered by pixels stained for GFAP in 7 patients at different ages. Several images were taken for each patient, n = 58 in total. **b.** Ezrin immunostaining intensity averaged in the astrocyte territorial domain and normalized to the immunostaining intensity of soma in 7 patients at different ages. Several cells were taken for each patient, n = 21 in total. **c.** Western blot: GFAP amount normalized to total protein amount in 14 patients at different ages. Number of blots is equal to the number of patients. **d,e.** same as c. but for ezrin (d) and glutamine synthetase (e, GS)

Empty circles are females, filled circles are males.

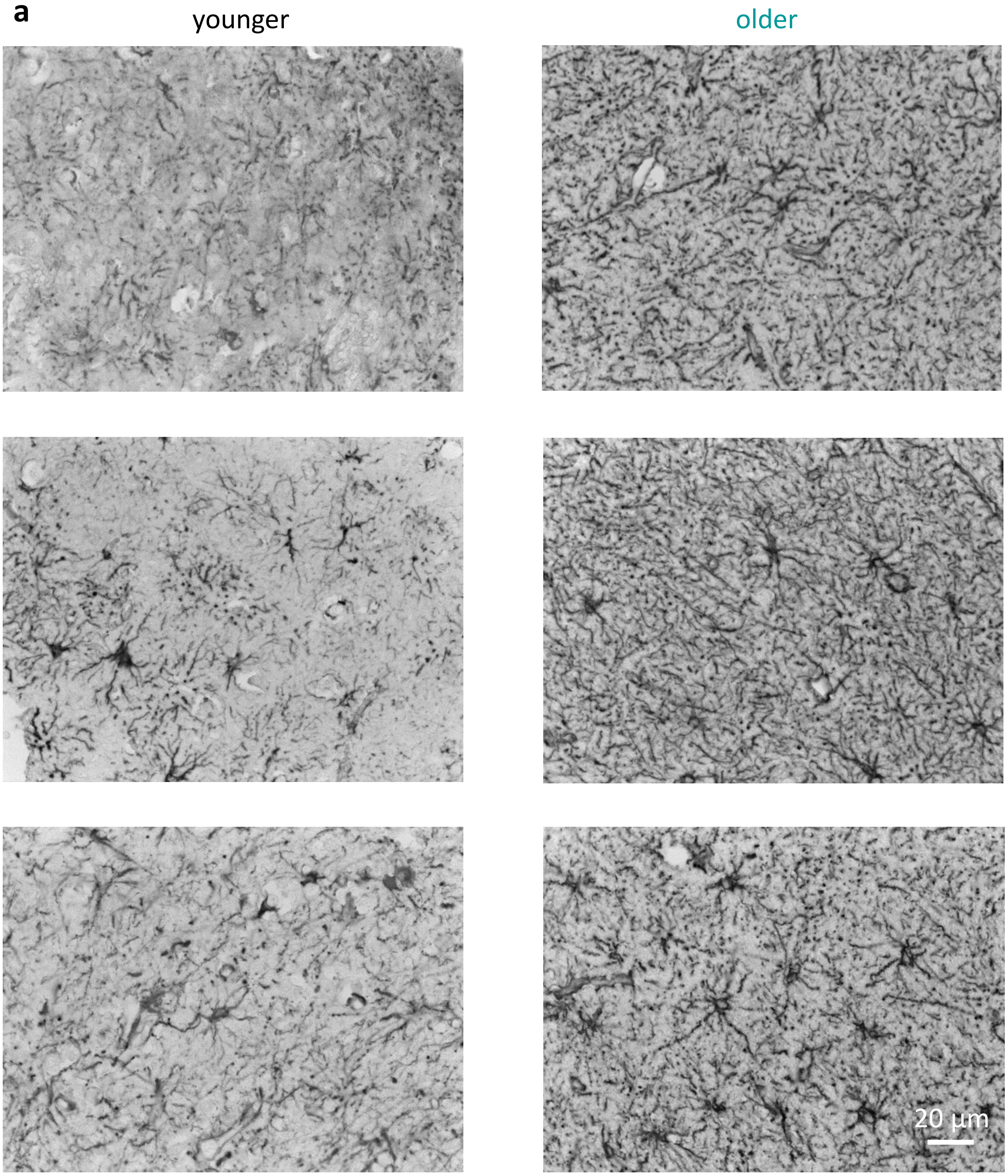

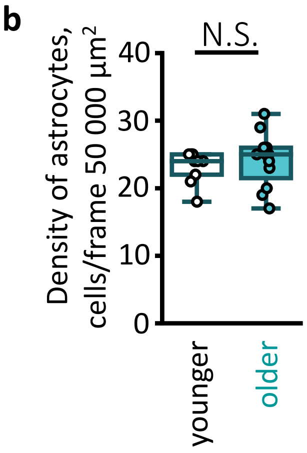

Supplementary Figure 6. Aging does not affect the density of GFAP-expressing astrocytes.

a. Representative GFAP immunostainings of cortical astrocytes in three younger adults (left column) and three older adults (right column). **b.** Summary plot showing no significant difference in the density of GFAP-positive astrocytes in two age groups ($p = 0.33$; younger adults: $N = 3$ people [$n = 9$ images]; older adults: $N = 4$ people [$n = 12$ images]).
Data are shown as box-and-whisker plots where the box is Q1 and Q3 with median, whiskers are the ranges within 1.5IQR. Empty boxes/circles – younger adults, filled boxes/circles – older adults. Mann-Whitney test. N.S. – $p > 0.05$.

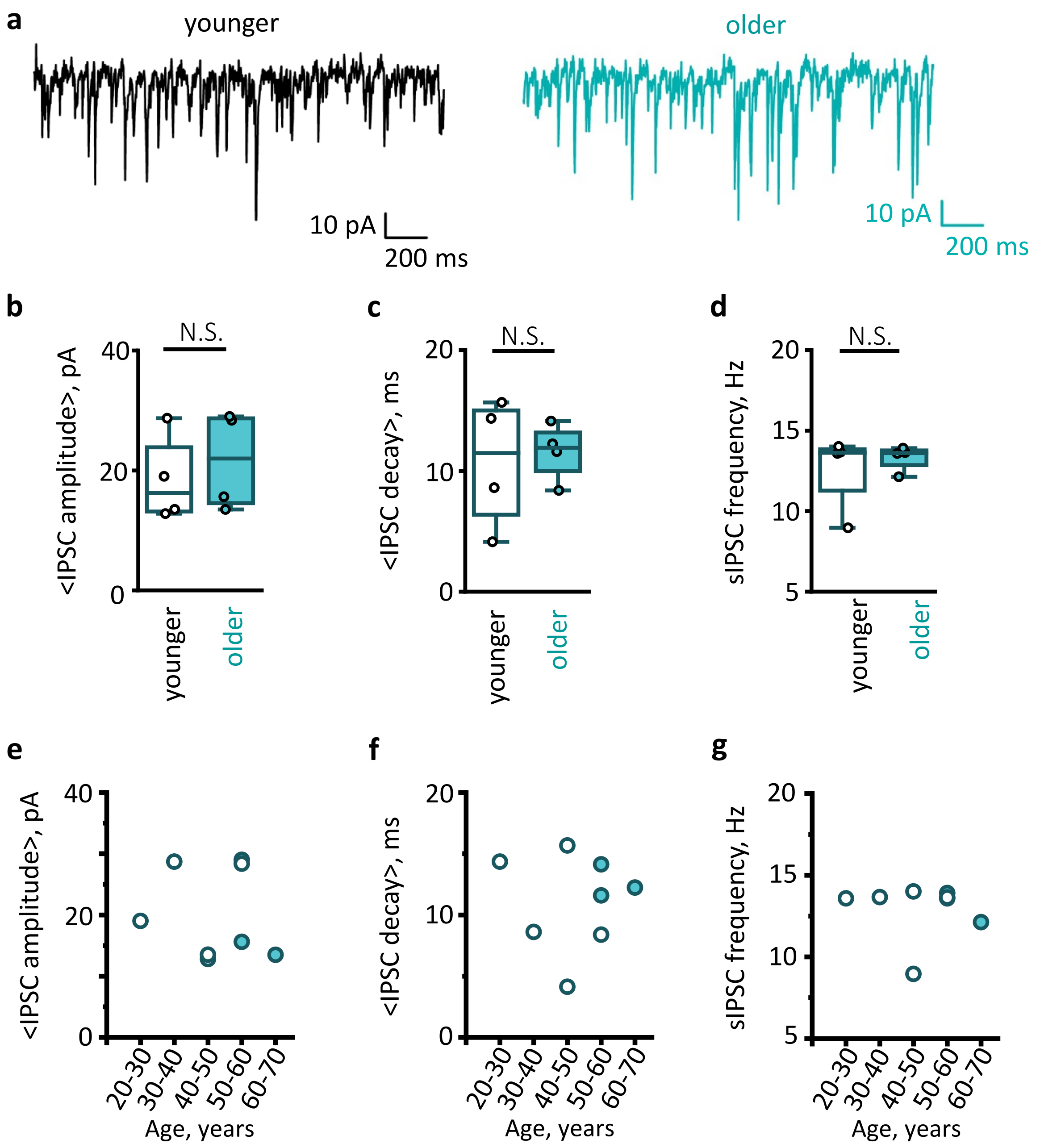

Supplementary Figure 7. Aging does not affect sIPSCs in cortical pyramidal neurons.

a. sIPSC recorded in voltage clamp mode in cortical pyramidal neurons. **b.** Mean amplitudes of sIPSCs in individual cells ($p = 0.67$), **c.** mean sIPSC decays ($p = 0.89$) and **d.** sIPSCs frequencies ($p = 0.89$) in two age groups (younger adults: N = 4 people; older adults: N = 4 people; cell number n = N). Data are shown as box-and-whisker plots where the box is Q1 and Q3 with median, whiskers are the ranges within 1.5IQR. Empty boxes/circles – younger adults, filled boxes/circles – older adults. Mann-Whitney test. N.S. – $p > 0.05$. **e,f,g.** same as on panels b,c,d for patients of different age groups. Empty circles are females, filled circles are males.
