# Supplementary material for: Mitochondrial malfunction and atrophy of astrocytes in the aged human cerebral cortex
Source: Nat Commun. 2023 Dec 16;14:8380. doi: 10.1038/s41467-023-44192-0 (PMC10725430; doi:10.1038/s41467-023-44192-0)
Supplement: Supplementary file 2 — Reporting Summary [file 41467_2023_44192_MOESM2_ESM.pdf]

Corresponding author(s): Alexey Semyanov, Alexei Verkhratsky

Last updated by author(s): Nov 21, 2023

Reporting Summary

Nature Portfolio wishes to improve the reproducibility of the work that we publish. This form provides structure for consistency and transparency in reporting. For further information on Nature Portfolio policies, see our Editorial Policies and the Editorial Policy Checklist.

Statistics

For all statistical analyses, confirm that the following items are present in the figure legend, table legend, main text, or Methods section.

n/a	Confirmed	
☐	☒	The exact sample size (*n*) for each experimental group/condition, given as a discrete number and unit of measurement
☐	☒	A statement on whether measurements were taken from distinct samples or whether the same sample was measured repeatedly
☐	☒	The statistical test(s) used AND whether they are one- or two-sided<br>*Only common tests should be described solely by name; describe more complex techniques in the Methods section.*
☒	☐	A description of all covariates tested
☒	☐	A description of any assumptions or corrections, such as tests of normality and adjustment for multiple comparisons
☐	☒	A full description of the statistical parameters including central tendency (e.g. means) or other basic estimates (e.g. regression coefficient) AND variation (e.g. standard deviation) or associated estimates of uncertainty (e.g. confidence intervals)
☐	☒	For null hypothesis testing, the test statistic (e.g. *F*, *t*, *r*) with confidence intervals, effect sizes, degrees of freedom and *P* value noted<br>*Give P values as exact values whenever suitable.*
☒	☐	For Bayesian analysis, information on the choice of priors and Markov chain Monte Carlo settings
☒	☐	For hierarchical and complex designs, identification of the appropriate level for tests and full reporting of outcomes
☒	☐	Estimates of effect sizes (e.g. Cohen's *d*, Pearson's *r*), indicating how they were calculated

Our web collection on statistics for biologists contains articles on many of the points above.

Software and code

Policy information about availability of computer code

Data collection	The data were collected with standard software supplied with respective equipment, except electrophysiology data recorded with WinWCP v5.2.5 software (University of Strathclyde, UK).
Data analysis	Raman spectra were analyzed with the open-source software Pyraman (DOI: 10.5281/zenodo.10151752)<br>Electrophysiological recordings were analyzed with the Clampfit 10.4 software (Molecular Devices, USA).<br>For astrocytes morphometry, we used custom code Image-funcut (DOI: 10.5281/zenodo.10151748).<br>Astrocyte branches were traced with the Simple neurite tracer plugin (https://github.com/morphonets/SNT) for ImageJ version 1.53 (NIH, Bethesda, USA).<br>The protein bands' intensity was quantified gel analyzer option of mageJ version 1.53 (NIH, Bethesda, USA)

For manuscripts utilizing custom algorithms or software that are central to the research but not yet described in published literature, software must be made available to editors and reviewers. We strongly encourage code deposition in a community repository (e.g. GitHub). See the Nature Portfolio guidelines for submitting code & software for further information.

Data

Policy information about availability of data

All manuscripts must include a data availability statement. This statement should provide the following information, where applicable:

- Accession codes, unique identifiers, or web links for publicly available datasets
- A description of any restrictions on data availability
- For clinical datasets or third party data, please ensure that the statement adheres to our policy

All data generated or analyzed during this study are included in the manuscript and supporting files.

Human research participants

Policy information about studies involving human research participants and Sex and Gender in Research.

Reporting on sex and gender	The samples were collected from both sexes. The gender information was not collected. Due to the limited sample size, statistical comparison of males and females was impossible, and the results were put together. Individual recordings from males and females are presented in supplementary figures and marked in Supporting files.
Population characteristics	The specimens of neocortical tissue were obtained during tumor resection from 42 patients in the range of ages from 18 to 71 years.
Recruitment	The patients undergoing tumor resection were routinely asked for informed consent to use their access tissue for research. Such methods or recruitment may introduce self-selection bias since volunteers may differ from those who chose not to participate in research. However, the absolute majority (over 90%) of the patients agreed to participate in the research. Thus, we don't assume significant self-selection bias in the current study. However, since the study was performed on the patients, the results may differ from those of the healthy population and reflect the effect of the tumor on the brain tissue.
Ethics oversight	Ethical Committee of the Privolzhsky Federal Research Medical Centre of the Ministry of Health of the Russian Federation

Note that full information on the approval of the study protocol must also be provided in the manuscript.

Field-specific reporting

Please select the one below that is the best fit for your research. If you are not sure, read the appropriate sections before making your selection.

[x] Life sciences [ ] Behavioural & social sciences [ ] Ecological, evolutionary & environmental sciences

For a reference copy of the document with all sections, see nature.com/documents/nr-reporting-summary-flat.pdf

Life sciences study design

All studies must disclose on these points even when the disclosure is negative.

Sample size	No statistical method was used to determine the sample size. The sample size was determined by the availability of the access tissue.
Data exclusions	No data were excluded from the analysis.
Replication	To ensure the reproducibility of experimental findings, we share processed experimental data and custom codes used in this study.
Randomization	Samples were divided into two groups: younger (18 - 50 years) and older (51 - 71 years) adult patients. Covariates control was not performed in our study.
Blinding	The experimenters were not blinded. The experimenter was informed in advance of the type of planned neurosurgery, including the patient's age, to apply for tissue collection knowingly.

Reporting for specific materials, systems and methods

We require information from authors about some types of materials, experimental systems and methods used in many studies. Here, indicate whether each material, system or method listed is relevant to your study. If you are not sure if a list item applies to your research, read the appropriate section before selecting a response.

Materials & experimental systems

n/a	Involved in the study
[ ]	[x] Antibodies
[x]	[ ] Eukaryotic cell lines
[x]	[ ] Palaeontology and archaeology
[x]	[ ] Animals and other organisms
[x]	[ ] Clinical data
[x]	[ ] Dual use research of concern

Methods

n/a	Involved in the study
[x]	[ ] ChIP-seq
[x]	[ ] Flow cytometry
[x]	[ ] MRI-based neuroimaging

Antibodies

Antibodies used

Raman microspectrometry
GFAP, Abcam, ab7260, polyclonal, rabbit
Secondary Cy™2 AffiniPure donkey anti-rabbit IgG (H+L), Jackson Immunoresearch, 711-225-152, polyclonal
NeuN, Novus Biologicals, NBP2-10491, chicken, polyclonal
Secondary Cy5 AffiniPure donkey anti-chicken IgG (H+L), Jackson Immunoresearch, polyclonal

Immunocytochemical analysis
Ezrin, Diagnostic Biosystems, Mob380, monoclonal, clone 3C12, mouse
GFAP, Biocare Medical, CM065, monoclonal, clone GA-5, mouse

Western blotting
GFAP, Antibodies-online, ABIN3044350, polyclonal, rabbit
Peroxidase AffiniPure Donkey Anti-Mouse IgG (H+L), Jackson immunoresearch, 715-035-150, polyclonal
Ezrin, Antibodies-online, ABIN5542456, monoclonal, clone 6F1A9, mouse
Goat Anti-Rabbit IgG H&L (HRP), Abcam, ab6721, polyclonal
GS, Synaptic Systems, 367 005, polyclonal, guinea pig
Peroxidase AffiniPure Donkey Anti-Guinea Pig IgG (H+L), Jackson Immunoresearch, 706-035-148, polyclonal

Validation

Validation information and relevant references are provided on manufacturer's websites
GFAP, Abcam, ab7260, polyclonal, rabbit
https://www.abcam.com/products/primary-antibodies/gfap-antibody-ab7260.html
RRID:AB_305808
Secondary Cy™2 AffiniPure Donkey Anti-Rabbit IgG (H+L), Jackson Immunoresearch, 711-225-152, polyclonal
https://www.jacksonimmuno.com/catalog/products/711-225-152
RRID: AB_2340612
NeuN, Novus Biologicals, NBP2-10491, chiken, polyclonal
http://www.novusbio.com/products/rbfox3-neun-antibody_nbp2-10491
RRID: AB_3075454
Secondary Cy5 AffiniPure donkey anti-chicken IgG (H+L), Jackson Immunoresearch, , polyclonal
https://www.jacksonimmuno.com/catalog/products/703-175-155
RRID: AB_2340365
Ezrin, Diagnostic Biosystems, Mob380, monoclonal, clone 3C12, mouse
https://dbiosys.com/product/ezrin-p81-cytovillin/?attribute_pa_volume=concentrated-1-ml-2
RRID: AB_3075452
GFAP, Biocare Medical, CM065, monoclonal, clone GA-5, mouse
https://biocare.net/product/glial-fibrillary-acidic-protein-antibody-2/
RRID: AB_3075453
GFAP - Antibodies-online, ABIN3044350, polyclonal, rabbit
https://www.antibodies-online.com/antibody/3044350/anti-Glial+Fibrillary+Acidic+Protein+GFAP+AA+417-432,+C-Term+antibody/
RRID: AB_3075450
Secondary Peroxidase AffiniPure Donkey Anti-Mouse IgG (H+L), Jackson immunoresearch, 715-035-150, polyclonal
https://www.jacksonimmuno.com/catalog/products/715-035-150
RRID: AB_2340770
Ezrin - Antibodies-online, ABIN5542456, monoclonal, clone 6F1A9, mouse
https://www.antibodies-online.com/antibody/5542456/anti-Ezrin+EZR+AA+292-464+antibody/
RRID: AB_3075451
Secondary Goat Anti-Rabbit IgG H&L (HRP), Abcam, ab6721, polyclonal
https://www.abcam.com/products/secondary-antibodies/goat-rabbit-igg-hl-hrp-ab6721.html
RRID:AB_955447
GS - Synaptic Systems, 367 005, polyclonal, guinea pig
https://www.sysy.com/product/367005
RRID:AB_2620128
Secondary Peroxidase AffiniPure Donkey Anti-Guinea Pig IgG (H+L), Jackson Immunoresearch, 706-035-148, polyclonal
https://www.jacksonimmuno.com/catalog/products/706-035-148

RRID: AB_2340447
